# A reduction of Syndecan-4 in macrophages promotes atherosclerosis by aggravating the proinflammatory capacity of macrophages

**DOI:** 10.1186/s12967-022-03505-5

**Published:** 2022-07-16

**Authors:** Jiaxin Hu, Ying Zhang, Liaoping Hu, Haiting Chen, Han Wu, Jianzhou Chen, Jun Xie, Biao Xu, Zhonghai Wei

**Affiliations:** Department of Cardiology, MOE Key Laboratory of Model Animal for Disease Study, Nanjing Drum Tower Hospital, The Affiliated Hospital of Nanjing University Medical School, Nanjing University, Nanjing, 210061 China

**Keywords:** Syndecan-4, Atherosclerosis, Macrophage, Ox-LDL, ApoE^−/−^

## Abstract

**Background:**

Cardiovascular diseases (CVDs) are a significant cause of mortality worldwide and are characterized by severe atherosclerosis (AS) in patients. However, the molecular mechanism of AS formation remains elusive. In the present study, we investigated the role of syndecan-4 (SDC4), a member of the syndecan family, in atherogenesis.

**Methods and Results:**

The expression of SDC4 decreased in mouse severe AS models. Moreover, knockout of SDC4 accelerated high-cholesterol diets (HCD)-induced AS in ApoE^−/−^ mice. Mechanistically, the decrease of SDC4 increased macrophage proinflammatory capacity may be through the PKCα-ABCA1/ABCG1 signaling pathway.

**Conclusion:**

These findings provide evidence that SDC4 reduction links macrophages and inflammation to AS and that SDC4 in macrophages provides a therapeutic target for preventing AS formation.

## Introduction

Coronary heart disease(CHD) is a global problem, which is the leading cause of cardiac death. Nowadays, there have been 11 million CHD patients and the annual mortality is 330/100 thousand persons in China, which has surpassed the mortality of tumors or other chronic diseases [1]. Similarly, approximately 64% of cardiac death is attributed to CHD in Western countries [2]. The optimal medical therapy and wide performance of percutaneous coronary intervention(PCI) have reduced the mortality of acute coronary syndrome(ACS) remarkably but can not reduce the all-cause death of stable coronary disease(SCD) [3]. One of the most important causes is that atherosclerosis(AS) is a systemic problem whereas PCI just solves a local problem of the pan vascular disease. So far, we still lack the effective measurements to reverse the plaque, which necessitate a profound understanding of the mechanism of plaque formation and progression.

AS is a chronic, low-grade inflammatory course, which is characterized by the infiltration of the inflammatory cells, deposition of oxidized low-density lipoprotein (oxLDL), the uncontrolled uptake of oxLDL by macrophages, and the subsequent formation of atheromatous plaques [4, 5]. During the numerous inflammatory cells, macrophage has played a pivotal role in atherogenesis. Obtaining deeper insight into the molecular mechanisms in the macrophages during atherogenesis is essential for identifying novel therapeutic targets.

Syndecan-4 (SDC4) is a member of the syndecan family, which is the major cell-surface heparan sulfate proteoglycans receptors widely expressed in the various tissues. It has a protein core and is covalently linked with linear chains of polysaccharides. The extracellular domain of SDC4 mediates the extracellular interaction, while the intracellular domain functions as the trigger of various signal processes. SDC4 plays numerous biological roles such as cell migration, proliferation, adhesion, endocytosis, tissue repair and regeneration, cell–matrix interactions, and matrix remodeling [6–9]. Physiologically, the ectodomain of SDC4 could be shed from the cells and the cells would re-express the receptor for the dynamic balance [10, 11]. In case of various stress, such as ischemia, hypoxia, and infection, the expression of SDC4 receptors will be upregulated [12, 13]. Meanwhile, the ectodomains also shed off substantially [14]. Several studies have demonstrated that ectodomain shedding of the SDC4 receptors plays an essential role in regulating inflammation in different pathophysiological conditions [15–17]. It has been accepted that macrophage-mediated vascular inflammatory responses are a critical factor in the formation of atherosclerosis [18–20]. Whether the SDC4 on the macrophage surface modulated the biological behavior of the macrophages in atherogenesis remains unclear.

Hence, this study explored the role of SDC4 on the surface of macrophages in AS initiation and progression. The results revealed that a reduction of SDC4 in macrophages promotes atherosclerosis by aggravating the proinflammatory capacity of macrophages. Our study, therefore, provided insight into developing a new drug target for the clinical treatment of AS.

## Methods

### Animal experimental protocol

The study protocol with animals was approved by the Institutional Ethics Committee of Nanjing Drum Tower Hospital (Approval No.2019-190-01) and performed by following the ARRIVE guidelines and recommendations developed by the National Centre for the Replacement, Refinement, and Reduction of Animals in Research (NC3Rs).

ApoE^−/−^ mice were purchased from the Model Animal Research Centre of Nanjing University (8 weeks old). SDC4^−/−^ mice with a C57BL/6 background were purchased from Jackson Laboratory. ApoE^−/−^ mice and SDC4^−/−^ mice were crossbred to obtain SDC4^−/−^ApoE^−/−^ mice. We provided standard feed, free water, and food to the animals and kept them in an environment with controlled temperature (22 ± 1 °C) and humidity (65–70%) and a 12 h light/dark cycle. The mice were fed high-cholesterol diets(HCD) (3% cholesterol, 0.2% bile salts, 20% lard, 10% white sugar, and high protein feed)for 12 weeks to induce the severe AS model, and the control mice were fed equal regular chow diets. At the end of the study, all animals were anesthetized by isoflurane inhalation (1.5–2%) and euthanized by cervical dislocation.

### Histological analysis

Following 12 weeks of modeling, the mice were sacrificed. The blood was cleaned with normal saline by perfusing the left ventricle. The vessels from the aortic arch to the iliac artery segment were dissected and then fixed in 4% formalin. The aortic arch segment and heart-aortic root samples were gradually dehydrated and embedded in paraffin and OCT respectively, and 5 µm thick sections were prepared.

For H.E. staining, hematoxylin and eosin staining were used to analyze the plaque burden in aortic root sections.

Masson staining was used to analyze the collagen content of aortic root sections.

For oil red O staining, after drying frozen sections at room temperature for 30 min, they were incubated with 0.5% oil red stain for 30 min. The sections were then washed with 60% isopropanol for 10 min and photographed under a microscope (Leica, TCSSP8).

For immunofluorescence staining, the deparaffinized tissue slides were boiled in citrate buffer solution at 100℃ for 1 h to extract the antigen, then were blocked in 1% fetal bovine serum at room temperature for 1 h, and then incubated with primary antibody at 4 ℃ overnight. Washing with PBS for 2–3 times, 5 min each time, then incubate the secondary antibody at room temperature for 1 h, wash with PBS for 2–3 times, DAPI staining, apply at room temperature for 10 min, wash with PBS twice in the absence of light. The anti-fluorescence quencher was blocked and photographed by a confocal microscope(Leica, TCSSP8, objective 200x)immediately.

### Cell culture and treatment

Induction of macrophages differentiation from mononuclear cells of aortic arch plaque tissue: aseptically, plaque tissue of mice(SDC4^−/−^ApoE^−/−^ and ApoE^−/−^ mice) were removed, and extracted macrophages as previously described [21]. The obtained cells were inoculated in six-well plates with 3 ml of culture medium. The culture medium included RPMI 1640 medium containing 10% fetal bovine serum with 100 U/ml penicillin, 0.1 mg/ml streptomycin, 2 nmol L-glutamine, and 50 ng/ml GM-CSF. Following this, the medium was changed every three days for a total period of 3 weeks. Then, 100 mg/L oxLDL was added to the culture medium and incubated for 48 h before being rinsed three times with DPBS and stained with Oil Red O.

### ***Recombinant lentivirus vector assembly and transduction into SDC4***^***−/−***^***ApoE***.^***−/−***^***macrophages***

In this study, we constructed a GV358 vector (Ubi-MCS-3FLAG-SV40-EGFP-IRES-puromycin) carrying SDC4 and was co-transfected with lentivirus backbone plasmid into 293 T cells to produce the recombinant lentivirus vector LV-SDC4. We generated empty viruses with another GV358 vector without SDC4 cDNA as controls. SDC4^−/−^ApoE^−/−^macrophages were cultured overnight in the six-well plate at the density of 3–5 × 10^4^ cells/ml. Lentivirus (1 × 10 ~ 9 TU/ml) was diluted with 1 ml complete medium containing HitransG P (1 mol/ml, GeneChem, China) and added to the cells. After transfection at 37 ℃ for 24 h, the virus culture medium was removed, washed 2–3 times with PBS, and replaced with the fresh virus-free medium. After continuous culture for 72 h, GFP-positive cells were identified as successfully transfected cells. Next, puromycin (5 ug/ml) was used to kill the negative cells, and the successfully transfected cells will survive.

### Western blot analysis

Protein was extracted from cells or tissues using RIPA buffer with protease inhibitors (Sigma). Then, 5 × protein loading buffer was added to the sample at a protein: buffer ratio of 4:1, and the sample was heated in a metal bath at 60 °C for 10 min. Protein electrophoresis was performed using the TGX FastCast acrylamide kit (BIO) and then transferred to PVDF membranes (Millipore, Bedford, MA, America). After blocking in 5% skim milk for 2 h, the membrane was incubated with primary antibody (1:1000 or 1:500) in a refrigerator at 4 °C overnight. GAPDH was used as the loading control. Next, the membrane was washed in (0.1%) (TBS-T) at least three times for 10 min each and incubated with horseradish peroxidation-enhanced secondary antibody (1:10,000) for 2 h. ECL Prime (G.E.) was used to test the protein bands according to the manufacturer's instructions. The antibodies used in the experiment were as follows: GAPDH (ProMab,20,035); ABCA1 (Abcam,ab66217); ABCG1 (Abcam,ab52617); and p-PKCα (Abcam, ab32502;PKCα (Abcam, ab32376).

### Enzyme-linked immunosorbent assay (ELISA)

The levels of TNF-α, MCP-1, IL-8, IL-6, IL-2, IL-1β, Cholesterol Ester(C.E.), Free cholesterol(F.C.), and total cholesterol(TC) were detected with specific ELISA kits (MultiSciences).

### Statistical analysis

Shapiro–Wilk test was used to determine whether the data were normally distributed. The data were presented as mean ± standard deviation (SD) when they were normally distributed or median and interquartile range(IQR) when they were skewed. Student t-test was used for comparison between two groups and one-way ANOVA was used for comparisons of multiple groups. Furthermore, the least significant difference(LSD) test was used for between-group comparisons when there were significant differences in the one-way ANOVA. P < 0.05 was considered significant (*p < 0.05, **p < 0.01, ***p < 0.001, ****p < 0.0001, ns = not significant). All the statistical analyses were performed by Graphpad Prism 8.02(GraphPad Software, La Jolla California USA).

## Results

### Reduced SDC4 expression in murine AS tissues

**Fig. 1:**
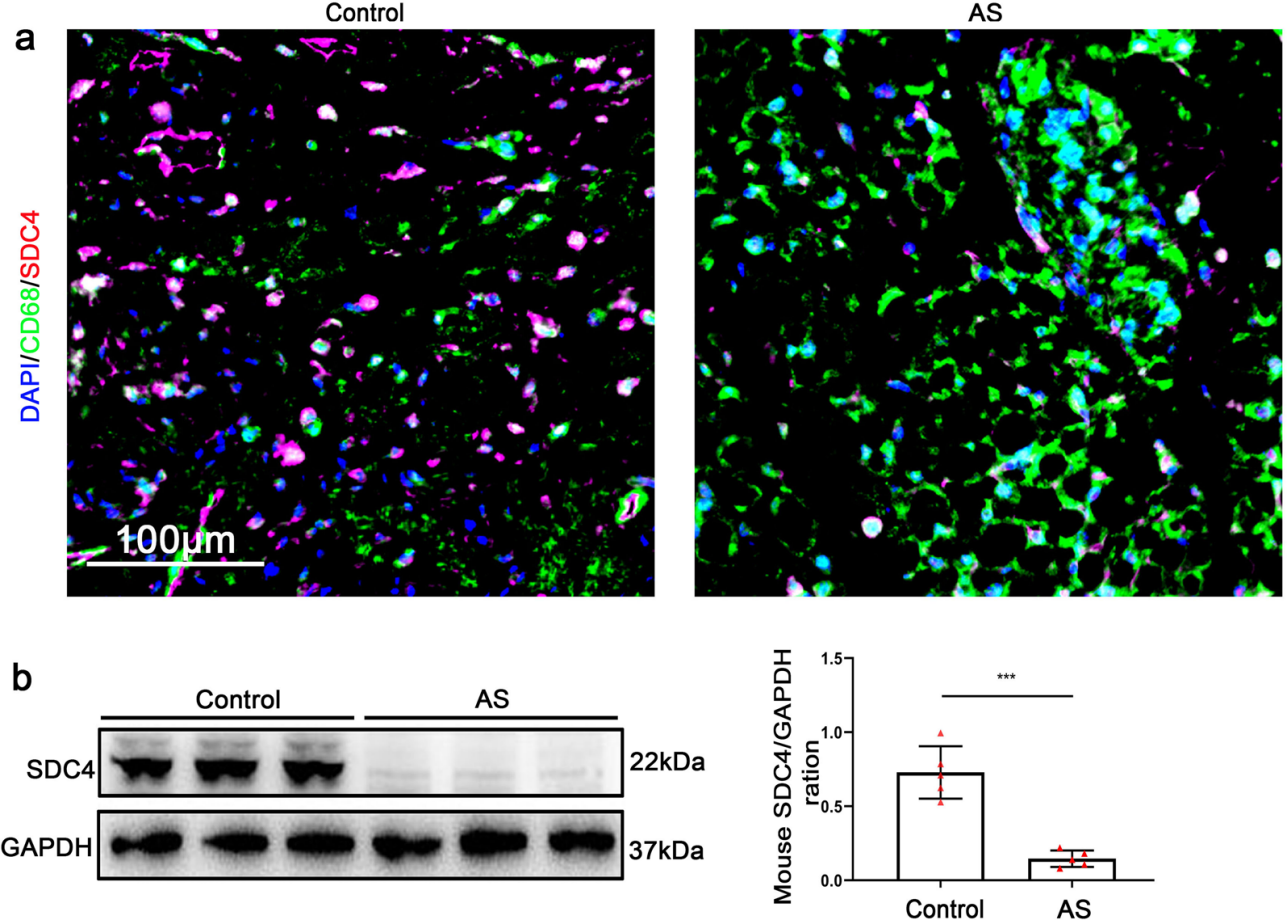
SDC4 was reduced in mouse AS tissues. **a** Representative immunofluorescence images of SDC4 in AS and control tissues, CD68(green), SDC4 (red), and DAPI (blue) (scale bars, 100 μm). **b** Representative western blots of SDC4 and densitometric analysis of AS and control tissues (n = 5)

We examined the expression of SDC4 in AS and normal mice tissues using immunofluorescence staining (Fig. 1a) and western blotting (Fig. 1b). As is shown in Fig. 1a, the level of SDC4 of macrophages is significantly lower in AS than in normal vascular tissues. Similarly, the western blotting also showed that the SDC4 expression was significantly lower in AS vascular tissues than in normal vascular tissues(Fig. 1b). These data demonstrated that SDC4 downregulation occurred during the development of AS.

### SDC4 gene deletion promotes atherogenesis in mice

**Fig. 2:**
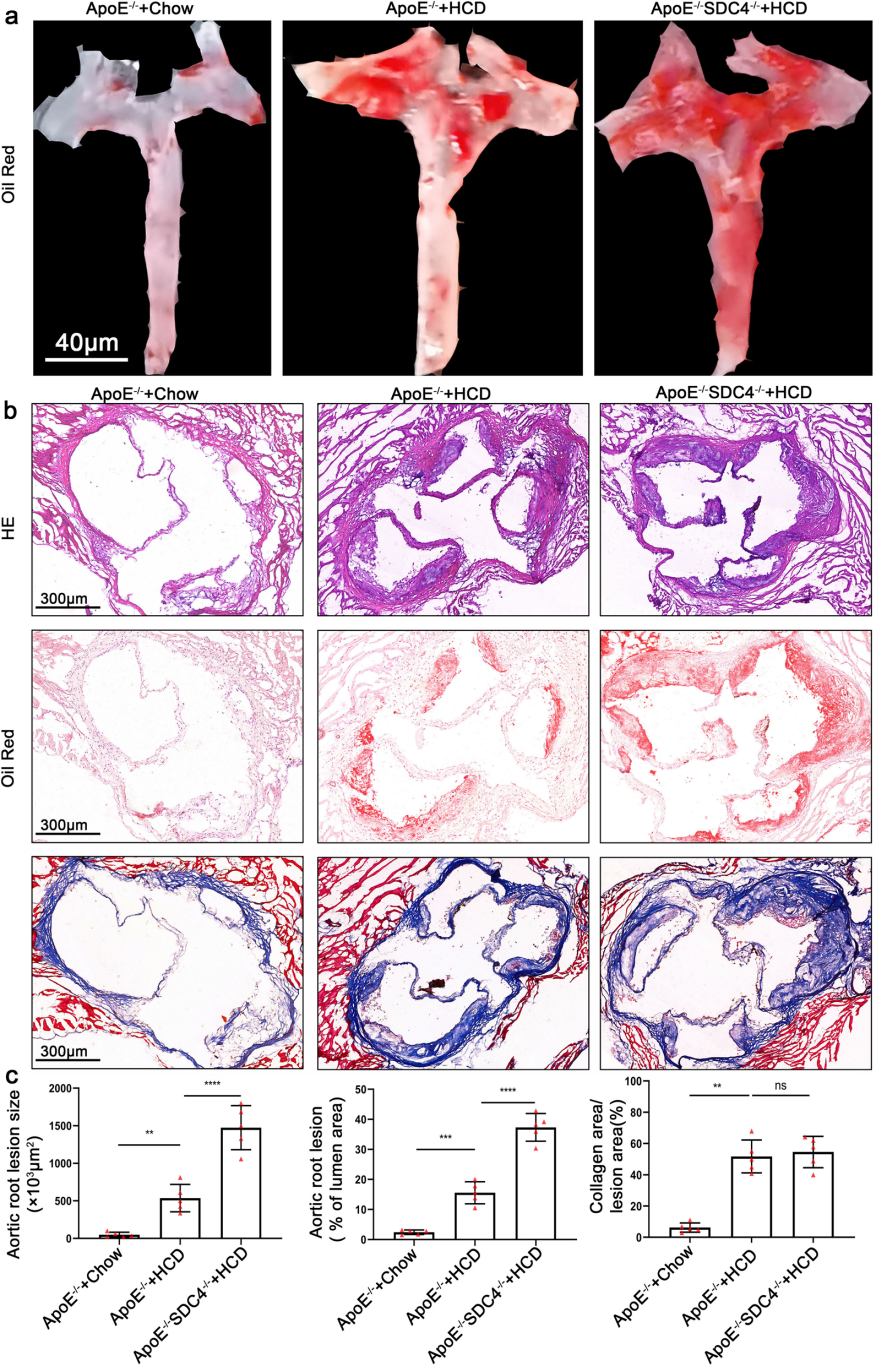
**a** Representative images of oil red O staining of the aortic arch of the mice, bar = 40 μm. **b** Representative images of H&E, oil red O, and Masson staining of aortic roots in ApoE^−/−^ and SDC4^−/−^ ApoE^−/−^ mice, bar = 300 μm. **c** Statistical analyses of the lesion area, lipid content, and collagen area in the aortic root,(n = 5 per group)

To explore the role of SDC4 in AS formation, we obtained SDC4^−/−^ApoE^−/−^ double-knockout (K.O.) mice by crossing ApoE^−/−^ mice with SDC4^−/−^ mice. Male mice are prone to develop atherosclerosis [22], so all mice used in this study were male. Here, 8 to 10-week-old male ApoE ^−/−^ mice (n = 10) and male SDC4^−/−^ApoE^−/−^ mice (n = 10) were fed a high-cholesterol diet for 12 weeks. After 12 weeks, all mice were sacrificed, and aortic root sections were used to quantify the plaque burden. Using oil red O staining of the aortic arch, we observed that SDC4^−/−^ApoE^−/−^ mice had more lipid accumulation than ApoE^−/−^ mice (Fig. 2a). Oil Red O and HE staining of aortic root sections showed that compared with ApoE^−/−^ mice, SDC4^−/−^ApoE^−/−^ mice had more lipid content and a more severe plaque burden (Fig. 2b, c). However, Masson staining showed that the collagen content has no significance in SDC4^−/−^ApoE^−/−^ mice compared with ApoE^−/−^ mice (Fig. 2b, c), which indicated that SDC4 knockdown does not affect plaque stability remarkably.

**Fig. 3:**
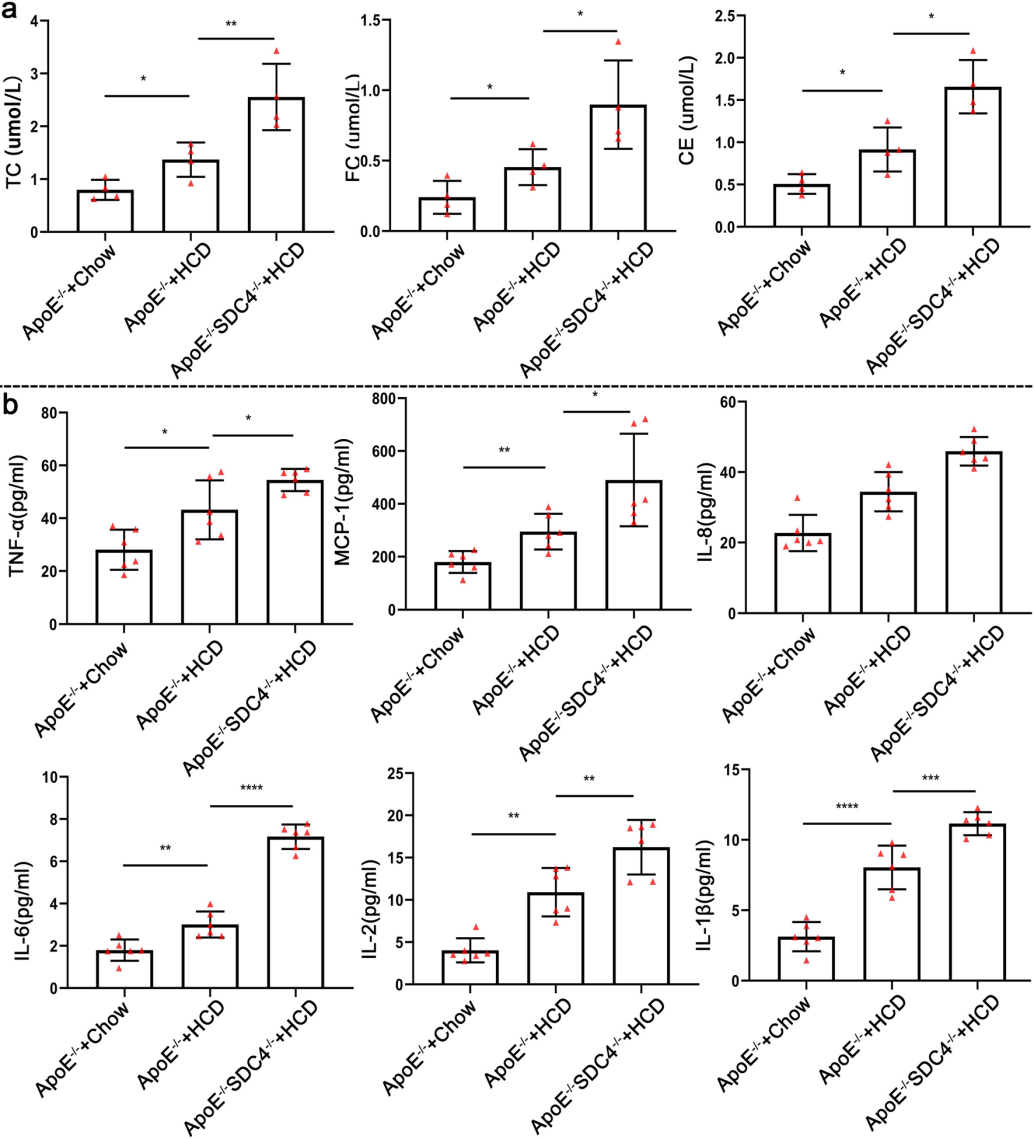
**a** The levels of CE, FC, and TC in the blood of ApoE^−/−^ and ApoE^−/−^ SDC4^−/−^ mice, (n = 4). **b** The levels of TNF-α, MCP-1, IL-8, IL-6, IL-2, and IL-1β in the vascular tissue of ApoE^−/−^and ApoE^−/−^SDC4^−/−^mice, (n = 6)

Interestingly, the blood levels of TC, CE, FC, and vascular tissue inflammatory factors (TNF-α, MCP-1, IL-8, IL-6, IL-2, IL-1β) were significantly higher in ApoE^−/−^SDC4^−/−^ mice than in ApoE^−/−^ mice (Fig. 3a, b). These results demonstrate that deleting the SDC4 gene in mice is associated with an increased lipid levels and inflammatory cytokines in AS formation.

### SDC4 deletion increased the level of inflammatory factors and reduced lipid transport in cultured primary macrophages

**Fig. 4:**
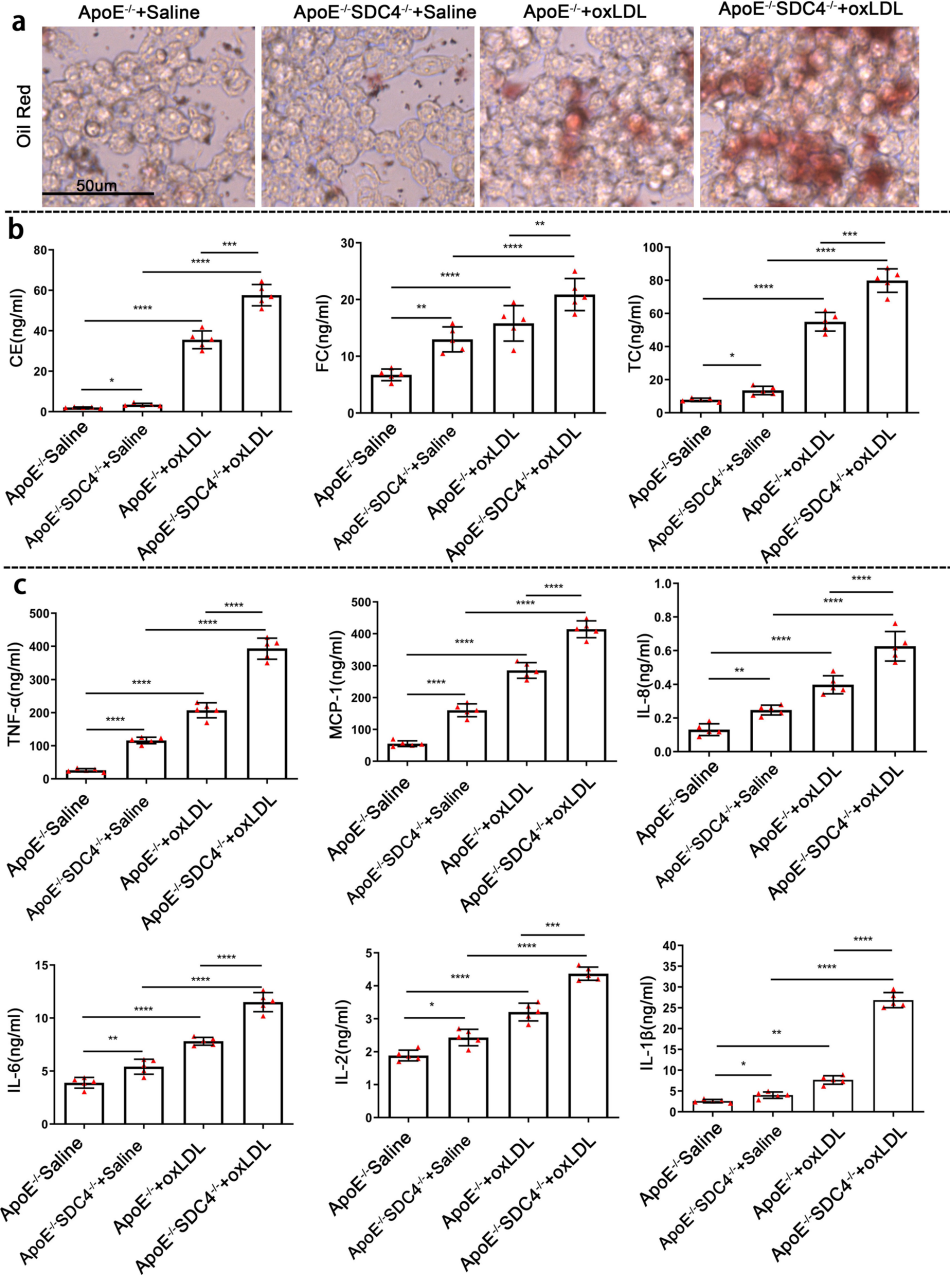
KO of SDC4 promoted the expression of inflammatory factors and reduced lipid transport in cultured macrophages. **a** Representative image of oil red O staining of ApoE^−/−^ and ApoE^−/−^ SDC4^−/−^ macrophages treated with or without ox-LDL. **b** The levels of CE, FC, and TC in the supernatants of ApoE^−/−^ + Saline, ApoE^−/−^SDC4^−/−^ + Saline, ApoE^−/−^ + ox-LDL, and ApoE^−/−^SDC4^−/−^ + ox-LDL cells were determined by ELISA (n = 5). **c** The levels of TNF-α, MCP-1, IL-8, IL-6, IL-2, and IL-1β in the supernatants of ApoE^−/−^ + Saline, ApoE^−/−^SDC4^−/−^ + Saline, ApoE^−/−^ + ox-LDL, and ApoE^−/−^SDC4^−/−^ + ox-LDL cells were determined by ELISA (n = 5)

Macrophages play a crucial role in the process of atherosclerosis [23], and we extracted macrophages from ApoE^−/−^ and SDC4^−/−^ApoE^−/−^ mice for the present study. The increased proinflammatory capacity of macrophages and impaired lipid transport are essential pathological mechanisms of atherosclerosis [24]. First, we divided the cells into four groups, ApoE^−/−^ + Saline, SDC4^−/−^ApoE^−/−^ + Saline, ApoE^−/−^ + oxLDL, SDC4^−/−^ApoE^−/−^ + oxLDL. ApoE^−/−^ + oxLDL and SDC4^−/−^ApoE^−/−^ + oxLDL cells were stimulated with oxLDL for 48 h, ApoE^−/−^ + Saline and SDC4^−/−^ApoE^−/−^ + Saline cells were stimulated with saline as control. Oil Red O staining showed that SDC4^−/−^ApoE^−/−^ + oxLDL cells had more lipid content than ApoE^−/−^ + oxLDL cells (Fig. 4a). Then, we collected the supernatants of ApoE^−/−^ + saline, SDC4^−/−^ApoE^−/−^ + saline, ApoE^−/−^ + oxLDL, and SDC4^−/−^ApoE^−/−^ + oxLDL cells and measured the levels of the inflammatory cytokines interleukin-1β (IL-1β), IL6, tumor necrosis factor-α (TNF-α), MCP-1, IL-8, IL-2, and C.E., F.C., TC. The results indicated that the levels of these molecules in SDC4^−/−^ApoE^−/−^ + saline cells were significantly higher than those in ApoE^−/−^ + saline cells. Furthermore, SDC4 deletion aggravated the expression of inflammatory cytokines induced by Ox-LDL (Fig. 4b, c). These data indicated that SDC4 silencing exacerbated inflammation and promoted the lipid deposition of macrophages.

### SDC4 regulates macrophages through the PKC-α-ABCA1/ABCG1 pathway

**Fig. 5:**
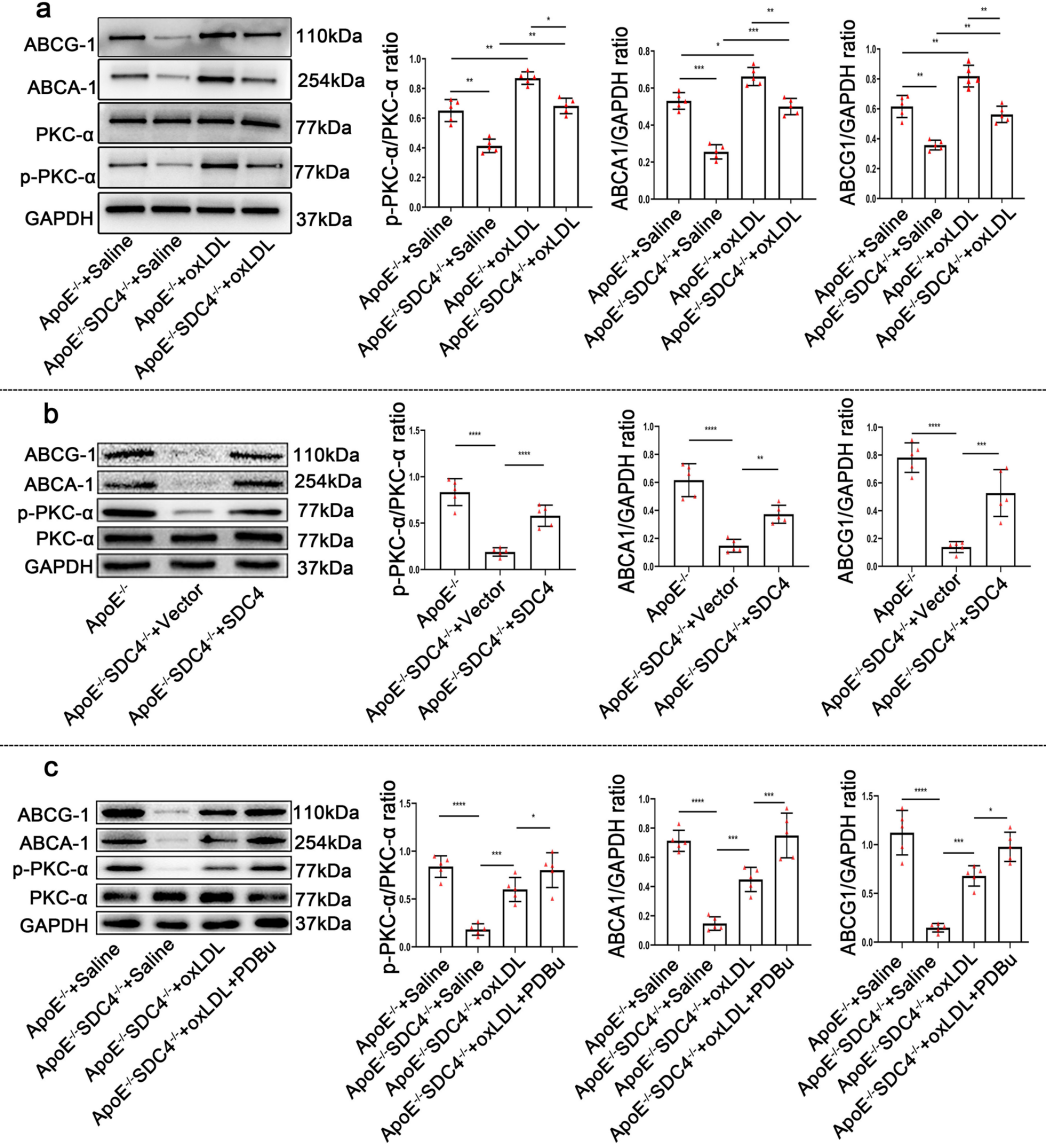
KO of SDC4 altered the PKC-α-ABCA1-ABCG1 pathway in cultured macrophages. **a** Representative western blots of PKC-α, ABCA1, ABCG1, and densitometric analysis of ApoE^−/−^ + Saline, ApoE^−/−^SDC4^−/−^ + Saline, ApoE^−/−^ + ox-LDL, and ApoE^−/−^SDC4^−/−^ + ox-LDL macrophages treated with or without ox-LDL for 48 h (n = 5). **b** Representative western blots of PKC-α, ABCA1, ABCG1 and densitometric analysis of ApoE^−/−^, ApoE^−/−^SDC4^−/−^ + Vector, ApoE^−/−^SDC4^−/−^ + SDC4 macrophages (n = 5). **c** Representative western blots of PKC-α, ABCA1, ABCG1 and densitometric analysis of ApoE^−/−^ + Saline, ApoE^−/−^SDC4^−/−^ + Saline, ApoE^−/−^SDC4^−/−^ + ox-LDL, and ApoE^−/−^SDC4^−/−^ + ox-LDL + PDBu macrophages (n = 5)

ATP-binding cassette transporter A1 and G1(ABCA1, ABCG1) are members of the ATP-binding cassette transporter family, which promotes the flow of intracellular lipids to the extracellular compartment and the reverse transport of intracellular lipids to HDL, thereby inhibiting foam cell formation [25]. External signaling cues have been demonstrated to promote ABCA1 expression by activating multiple signaling pathways, such as PKC, PKA, and Ca^2+^, increasing intracellular cholesterol efflux in several investigations [26, 27]. At the same time, mutations in ABCA1 result in a significant increase in atherosclerotic plaque load [28]. Because it is generally known that SDC4 may activate PKC independently [29], we focused on the PKC-ABCA1 pathway to learning more about how SDC4 regulates macrophages. The results showed that SDC4 deletion reduced the activity of PKCα, ABCA1, and ABCG1 compared with that in ApoE^−/−^ cells. In addition, the expression of PKCα, ABCA1, and ABCG1 was also lower in SDC4^−/−^ApoE^−/−^ + ox-LDL cells than in ApoE^−/−^ + ox-LDL cells (Fig. 5a). Then, we used lentivirus carrying SDC4 to transfect ApoE^−/−^SDC4^−/−^ cells and divided the cells into three groups, including ApoE^−/−^, ApoE^−/−^SDC4^−/−^ + vector, ApoE^−/−^SDC4^−/−^ + SDC4 and to observe the changes of PKC-**α**-ABCA1 pathway. The results showed that exogenous replenishment of SDC4 rescued the reduced activity of PKCα, ABCA1, and ABCG1 induced by SDC4 deletion (Fig. 5b). Further, we added ahorbol 12,13-dibutyrate (PDBu), an agonist of PKCα, to SDC4^−/−^ApoE^−/−^cells for 30 min [30]and then detected the expression of PKCα, ABCA1, and ABCG1 in ApoE^−/−^ + Saline, SDC4^−/−^ApoE^−/−^ + Saline and SDC4^−/−^ApoE^−/−^ + oxLDL, SDC4^−/−^ApoE^−/−^ + oxLDL + PDBu cells. As shown in Fig. 5c, PDBu stimulation also rescued the reduced activity of PKCα, ABCA1, and ABCG1 induced by SDC4 deletion.

**Fig. 6:**
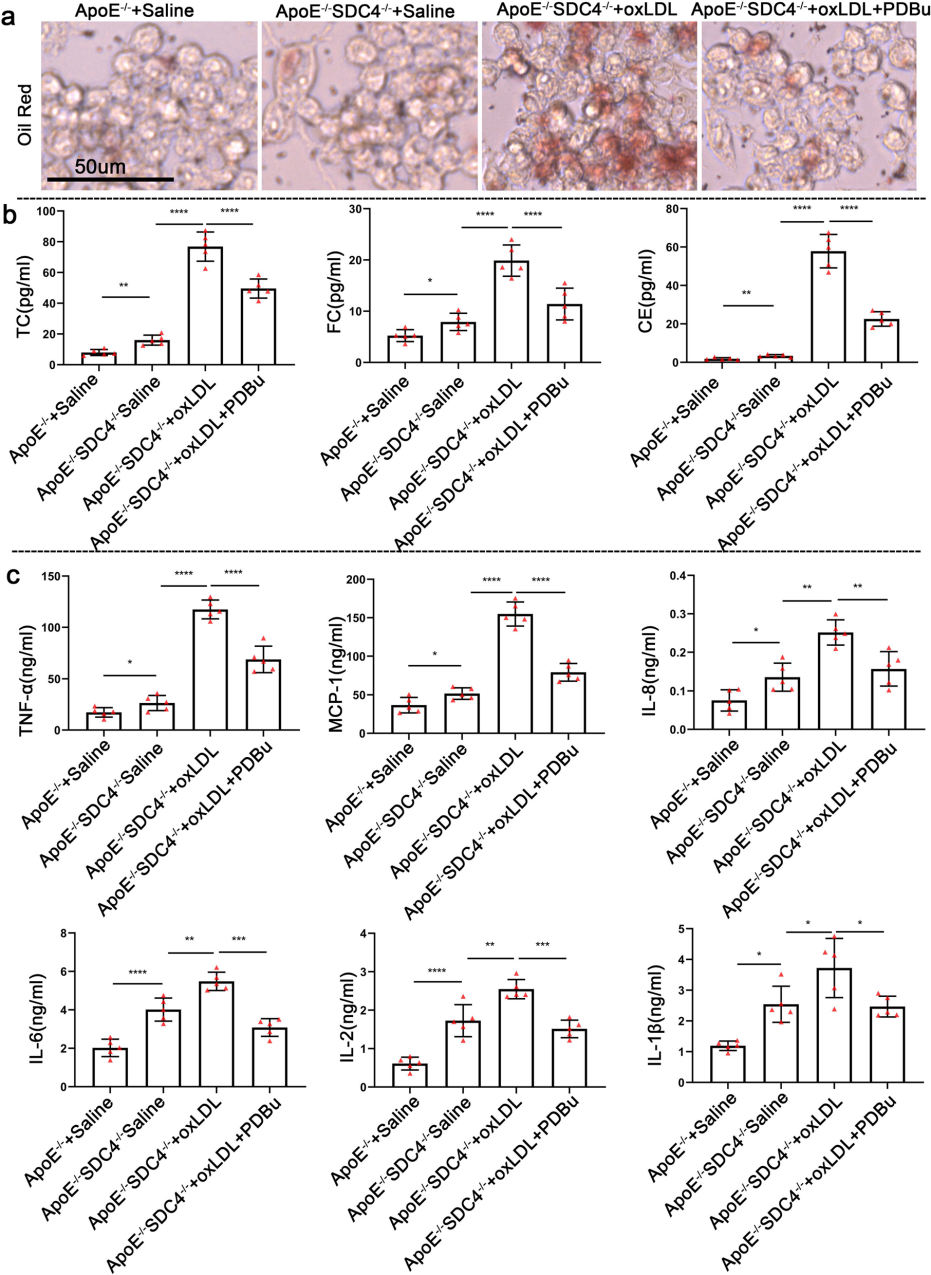
PDBu reduced the expression of inflammatory factors and increased lipid transport in cultured macrophages. **a** Representative image of oil red O staining of ApoE^−/−^ + Saline, ApoE^−/−^SDC4^−/−^ + Saline, ApoE^−/−^SDC4^−/−^ + ox-LDL, and ApoE^−/−^SDC4^−/−^ + ox-LDL + PDBu macrophages. **b** The levels of CE, FC, and TC in the supernatants of ApoE^−/−^ + Saline, ApoE^−/−^SDC4^−/−^ + Saline, ApoE^−/−^SDC4^−/−^ + ox-LDL, and ApoE^−/−^SDC4^−/−^ + ox-LDL + PDBu macrophages were determined by ELISA (n = 5). **c** The levels of TNF-α, MCP-1, IL-8, IL-6, IL-2, and IL-1β in the supernatants of ApoE^−/−^ + Saline, ApoE^−/−^SDC4^−/−^ + Saline, ApoE^−/−^SDC4^−/−^ + ox-LDL, and ApoE^−/−^SDC4^−/−^ + ox-LDL + PDBu macrophages were determined by ELISA (n = 5)

To further prove that SDC4 regulates macrophages through the PKCα-ABCA1/ABCG1 pathway. We detected the lipid transport and inflammatory factor secretion capability of macrophages after stimulation with PDBu. As shown in Fig. 6a, there were fewer oil red O-positive ApoE^−/−^SDC4^−/−^ + ox-LDL + PDBu cells than ApoE^−/−^SDC4^−/−^ + ox-LDL cells. Moreover, the C.E., F.C., and TC levels were also lower in ApoE^−/−^SDC4^−/−^ + ox-LDL + PDBu cells than in ApoE^−/−^SDC4^−/−^ + ox-LDL cells (Fig. 6b). In line with previous findings, the levels of TNF-α, MCP-1, IL-8, IL-6, IL-2, and IL-1β were significantly lower in ApoE^−/−^SDC4^−/−^ + ox-LDL + PDBu cells than in ApoE^−/−^SDC4^−/−^ + ox-LDL cells (Fig. 6c). These data demonstrated that PDBu reduced the proinflammatory capacity and promoted lipid transport in SDC4-deleted macrophages. These results indicated that SDC4 regulates macrophages may through the PKCα-ABCA1/ABCG1 pathway.

## Discussion

In the present study, we established that the loss of SDC4 exacerbated AS formation in a mouse model. We have various findings in this work. First, the expression of SDC4 in the vascular tissue was lower in the AS mouse model than in control mice. Second, SDC4 deletion in vivo exacerbated HCD-induced AS formation in ApoE^−/−^ mice. Third, SDC4 silencing extended the proinflammatory capability and decreased lipid efflux in macrophages via the PKCα-ABCA1/ABCG1 pathway.

SDC4 is a kind of non-specific transmembrane receptor and is ubiquitously expressed in various cells. It is associated with a wide variety of signal pathways and plays different physiological functions according to previous studies [31]. In the recent decade, it has been demonstrated that SDC4 could regulate the inflammatory response in different systems. Marie E. Strand et al. found the shedding ectodomains of SDC4 is able to recruit immune cells and alleviate cardiac inflammatory injury after exposure to the lipopolysaccharides(LPS) [32]. Similarly, Yoshinori Tanino et al. have revealed that SDC4 could inhibit the recruitment of neutrophils and limit the extent of lung injury in response to the LPS [17]. On the contrary, some other studies reported that SDC4 could promote allergic airway inflammation or initiate the inflammatory response of rheumatoid arthritis [15, 33]. In addition, Severine Brule et al. reported that Stromal cell-derived factor-1 (SDF-1), a chemokine, accelerated the shedding of syndecan-4 and syndecan-1 from HeLa cells and human primary macrophages [34]. These findings imply that SDC4 probably plays dual roles in different acute inflammatory settings or acts differently on diverse inflammatory cells.

Thus, we take great interest in the role of SDC4 plays in the chronic inflammatory course. Interestingly, we found the expression of SDC4 was down-regulated remarkably in the AS mice compared with the control mice, which has not been reported yet before. It is implied that the synthesis of SDC4 is possibly hampered by the pathophysiological processes, such as inflammation, oxidative stress, and cell apoptosis during plaque formation. Furthermore, down-regulation of SDC4 is not only the consequence of atherogenesis but also a promotor of atherogenesis. In the ApoE^−/−^ SDC4^−/−^mice, the size of aortic plaque lesions and lipid deposition in the plaque were both significantly increased. Meanwhile, the expression of diverse proinflammatory cytokines in the plaque lesions including IL-1β, IL-2, IL-8, TNF-α, IL-6, and MCP-1 were all elevated considerably, which implied a higher level of inflammatory activity in the vascular tissue in the absence of SDC4. Therefore, the acceleration of atherosclerosis in the ApoE^−/−^ SDC4^−/−^ mice was associated with the trigger of chronic inflammation.

The inflammation of atherogenesis usually involves different kinds of inflammatory cells. Macrophages are continuously recruited, aggregated, and take up more lipids in response to various inflammatory cytokines, which play a crucial role in atherogenesis. The pathological process is followed by inflicting foam cell accumulation while secreting more inflammatory mediators themselves and recruiting more macrophages and other inflammatory cells, ultimately leading to plaque formation and progression [5, 19, 35]. To identify how SDC4 modulates the biological function of macrophages during plaque formation, we extracted macrophages from ApoE^−/−^ mice and ApoE^−/−^ SDC4^−/−^ mice for in vitro study. Consequently, diverse proinflammatory cytokines secreted by ApoE^−/−^ SDC4^−/−^ macrophages were significantly elevated particularly in response to oxLDL stimulation. Thus, the increased proinflammatory capability of the macrophages promoted the higher inflammatory activity in the vascular lesions of the SDC4^−/−^ ApoE^−/−^mice. In this light, chronic inflammation and the expression of SDC4 affect reciprocally. That means the down- or up-regulation of SDC4 could be influenced by chronic inflammation and the expression level of SDC4 could also modulate the inflammatory activity. It has been demonstrated that heparan sulfate can bind diverse chemokines to maintain fine control of the chemokine gradient [36]. Hence, KO of SDC4 is thought to lose this balance and augment the extent of inflammation.

Besides, SDC4^−/−^ ApoE^−/−^ macrophages also manifested more lipid deposition in the cells, especially under stimulation of ox-LDL. As we know, the proinflammatory response is induced by lipid deposition. Nonetheless, how SDC4 regulates the inflammatory response of macrophages remains unclear. It has been revealed in the previous study that the absence of SDC4 can lead to a higher activity of RhoG at baseline and further make increased ruffling of the membrane, which promotes the micropinocytosis [37]. This finding could be a partial explanation for the accelerated lipid deposition in macrophages. Besides the increased uptake of lipid, the decrease of lipid efflux is another important aspect. ABCA1 and ABCG1 play a crucial role in the transport of the intracellular excess lipid to high-density lipoprotein (HDL) [38]. In vitro study, the phosphorylation of PKCα and the expression of ABCA1 and ABCG1 were decreased in the absence of SDC4 in comparison with the corresponding group no matter there was stimulation of oxLDL or not. Of note, the use of PDBu, the agonist of PKCα, in ApoE^−/−^ SDC4^−/−^ macrophages was able to upregulate the expression of ABCA1 and ABCG1, reduce the lipid deposition in the macrophages and inhibit the pro-inflammatory cytokines secreted from the macrophages. These findings have revealed that the biological function of the macrophages can be regulated via the SDC4-PKCα-ABCA1/ABCG1 signal pathway, which provides a potential mechanism for SDC4 in protecting the arteries against atherosclerosis.

In this study, there are some limitations. SDC4 and ApoE double-knockout mice were required to establish an HCD-induced AS model. Macrophage-specific SDC4 knockout would provide more definitive evidence. The present study used global SDC4 and ApoE knockout mice in the animal study, which caused limitations for research. In addition, AS is a complicated process, which involves some other inflammatory cells or immune cells besides macrophages, such as B and T lymphocytes, and dendritic cells. The regulation of SDC4 on the different inflammatory cells remains unclear and needs to be further investigated. Finally, we used male ApoE^−/−^ mice and induced a severe atherosclerosis model fed with HCD. Gender differences are strongly associated with cardiovascular disease, and it has been reported that the incidence of atherosclerosis tends to be lower in women than in men [39]. Therefore, the conclusion of this study is whether suitable for other models is still unknown.

## Conclusion

SDC4 plays a protective role during the process of atherogenesis. Loss of SDC4 will promote plaque formation remarkably by regulating the biological function of macrophages via the SDC4-PKCα-ABCA1/ABCG1 signal pathway. In this regard, SDC4 on the macrophages could serve as a novel target for the therapy of AS.

## Data Availability

Not applicable.

## Abbreviations

CVDs: Cardiovascular diseases
AS: Atherosclerosis; SCD4: syndecan-4
CHD: Coronary heart disease
ACS: Acute coronary syndrome
SCD: Stable coronary disease
ox-LDL: Oxidized low-density lipoprotein
HCD: High-cholesterol diets
CE: Cholesterol Ester
FC: Free cholesterol
TC: Total cholesterol
ABCA1: ATP-binding cassette transporter A1
ABCG1: ATP-binding cassette transporter G1
PCI: Percutaneous coronary intervention

## References

[1] Shengshou HU, Gao R, Liu L, Zhu M, Wang W, Wang Y, Zhaosu WU, Huijun LI, Dongfeng GU, Yang Y (2019). Summary of the 2018 report on cardiovascular diseases in China. Chin Circ J.

[2] Go AS, Mozaffarian D, Roger VL, Benjamin EJ, Berry JD, Borden WB, Bravata DM, Dai S, Ford ES, Fox CS (2013). Heart disease and stroke statistics–2013 update: a report from the American Heart Association. Circulation.

[3] Maron DJ, Hochman JS, Reynolds HR, Bangalore S, O'Brien SM, Boden WE, Chaitman BR, Senior R, Lopez-Sendon J, Alexander KP (2020). Initial invasive or conservative strategy for stable coronary disease. N Engl J Med.

[4] Zhang Y, Li Q, Welsh WJ, Moghe PV, Uhrich KE (2016). Micellar and structural stability of nanoscale amphiphilic polymers: implications for anti-atherosclerotic bioactivity. Biomaterials.

[5] Back M, Yurdagul A, Tabas I, Oorni K, Kovanen PT (2019). Inflammation and its resolution in atherosclerosis: mediators and therapeutic opportunities. Nat Rev Cardiol.

[6] Addi C, Presle A, Fremont S, Cuvelier F, Rocancourt M, Milin F, Schmutz S, Chamot-Rooke J, Douche T, Duchateau M (1941). The Flemmingsome reveals an ESCRT-to-membrane coupling via ALIX/syntenin/syndecan-4 required for completion of cytokinesis. Nat Commun.

[7] Valdivia A, Cardenas A, Brenet M, Maldonado H, Kong M, Diaz J, Burridge K, Schneider P, San Martin A, Garcia-Mata R (2020). Syndecan-4/PAR-3 signaling regulates focal adhesion dynamics in mesenchymal cells. Cell Commun Signal.

[8] Bellin RM, Kubicek JD, Frigault MJ, Kamien AJ, Steward RL, Barnes HM, Digiacomo MB, Duncan LJ, Edgerly CK, Morse EM (2009). Defining the role of syndecan-4 in mechanotransduction using surface-modification approaches. Proc Natl Acad Sci U S A.

[9] Herum KM, Lunde IG, Skrbic B, Louch WE, Hasic A, Boye S, Unger A, Brorson SH, Sjaastad I, Tonnessen T (2015). Syndecan-4 is a key determinant of collagen cross-linking and passive myocardial stiffness in the pressure-overloaded heart. Cardiovasc Res.

[10] Kim CW, Goldberger OA, Gallo RL, Bernfield M (1994). Members of the syndecan family of heparan sulfate proteoglycans are expressed in distinct cell-, tissue-, and development-specific patterns. Mol Biol Cell.

[11] Houston M, Julien MA, Parthasarathy S, Chaikof EL (2005). Oxidized linoleic acid regulates expression and shedding of syndecan-4. Am J Physiol Cell Physiol.

[12] Ozsoy HZ, Sivasubramanian N, Wieder ED, Pedersen S, Mann DL (2008). Oxidative stress promotes ligand-independent and enhanced ligand-dependent tumor necrosis factor receptor signaling. J Biol Chem.

[13] Julien MA, Wang P, Haller CA, Wen J, Chaikof EL (2007). Mechanical strain regulates syndecan-4 expression and shedding in smooth muscle cells through differential activation of MAP kinase signaling pathways. Am J Physiol Cell Physiol.

[14] Fitzgerald ML, Wang Z, Park PW, Murphy G, Bernfield M (2000). Shedding of syndecan-1 and -4 ectodomains is regulated by multiple signaling pathways and mediated by a TIMP-3-sensitive metalloproteinase. J Cell Biol.

[15] Cai P, Lu Z, Jiang T, Wang Z, Yang Y, Zheng L, Zhao J (2020). Syndecan-4 involves in the pathogenesis of rheumatoid arthritis by regulating the inflammatory response and apoptosis of fibroblast-like synoviocytes. J Cell Physiol.

[16] Vuong TT, Reine TM, Sudworth A, Jenssen TG, Kolset SO (2015). Syndecan-4 is a major syndecan in primary human endothelial cells in vitro, modulated by inflammatory stimuli and involved in wound healing. J Histochem Cytochem.

[17] Tanino Y, Chang MY, Wang X, Gill SE, Skerrett S, McGuire JK, Sato S, Nikaido T, Kojima T, Munakata M (2012). Syndecan-4 regulates early neutrophil migration and pulmonary inflammation in response to lipopolysaccharide. Am J Respir Cell Mol Biol.

[18] Koelwyn GJ, Corr EM, Erbay E, Moore KJ (2018). Regulation of macrophage immunometabolism in atherosclerosis. Nat Immunol.

[19] Tabas I, Bornfeldt KE (2016). Macrophage phenotype and function in different stages of atherosclerosis. Circ Res.

[20] Martinez GJ, Celermajer DS, Patel S (2018). The NLRP3 inflammasome and the emerging role of colchicine to inhibit atherosclerosis-associated inflammation. Atherosclerosis.

[21] Roake J, Marks E, Anderson N, Prebble H, Aamir R (2018). Induced macrophage activation in live excised atherosclerotic plaque. Immunobiology..

[22] Li J, Lin S, Vanhoutte PM, Woo CW, Xu A (2016). Akkermansia muciniphila protects against atherosclerosis by preventing metabolic endotoxemia-induced inflammation in apoe-/- mice. Circulation.

[23] Barrett TJ (2020). Macrophages in atherosclerosis regression. Arterioscler Thromb Vasc Biol.

[24] Moore KJ, Tabas I (2011). Macrophages in the pathogenesis of atherosclerosis. Cell.

[25] Attie AD (2007). ABCA1: at the nexus of cholesterol, HDL and atherosclerosis. Trends Biochem Sci.

[26] Liu Y, Tang C (2012). Regulation of ABCA1 functions by signaling pathways. Biochim Biophys Acta.

[27] Frambach S, de Haas R, Smeitink JAM, Rongen GA, Russel FGM, Schirris TJJ (2020). Brothers in arms: ABCA1- and ABCG1-mediated cholesterol efflux as promising targets in cardiovascular disease treatment. Pharmacol Rev.

[28] Ouimet M, Barrett TJ, Fisher EA (2019). HDL and reverse cholesterol transport. Circ Res.

[29] Panicker SR, Biswas I, Giri H, Cai X, Rezaie AR (2020). PKC (Protein Kinase C)-delta modulates AT (antithrombin) signaling in vascular endothelial cells. Arterioscler Thromb Vasc Biol.

[30] Ma D, Zheng B, Suzuki T, Zhang R, Jiang C, Bai D, Yin W, Yang Z, Zhang X, Hou L (2017). Inhibition of KLF5-Myo9b-RhoA pathway-mediated podosome formation in macrophages ameliorates abdominal aortic aneurysm. Circ Res.

[31] Simons M, Horowitz A (2001). Syndecan-4-mediated signalling. Cell Signal.

[32] Strand ME, Aronsen JM, Braathen B, Sjaastad I, Kvaloy H, Tonnessen T, Christensen G, Lunde IG (2015). Shedding of syndecan-4 promotes immune cell recruitment and mitigates cardiac dysfunction after lipopolysaccharide challenge in mice. J Mol Cell Cardiol.

[33] Polte T, Petzold S, Bertrand J, Schutze N, Hinz D, Simon JC, Lehmann I, Echtermeyer F, Pap T, Averbeck M (2015). Critical role for syndecan-4 in dendritic cell migration during development of allergic airway inflammation. Nat Commun.

[34] Brule S, Charnaux N, Sutton A, Ledoux D, Chaigneau T, Saffar L, Gattegno L (2006). The shedding of syndecan-4 and syndecan-1 from HeLa cells and human primary macrophages is accelerated by SDF-1/CXCL12 and mediated by the matrix metalloproteinase-9. Glycobiology.

[35] Hansson GK, Robertson AK, Soderberg-Naucler C (2006). Inflammation and atherosclerosis. Annu Rev Pathol.

[36] Tanino Y, Coombe DR, Gill SE, Kett WC, Kajikawa O, Proudfoot AE, Wells TN, Parks WC, Wight TN, Martin TR, Frevert CW (2010). Kinetics of chemokine-glycosaminoglycan interactions control neutrophil migration into the airspaces of the lungs. J Immunol.

[37] Elfenbein A, Lanahan A, Zhou TX, Yamasaki A, Tkachenko E, Matsuda M, Simons M (2012). Syndecan 4 regulates FGFR1 signaling in endothelial cells by directing macropinocytosis. Sci Signal.

[38] Ye D, Lammers B, Zhao Y, Meurs I, Van Berkel TJ, Van Eck M (2011). ATP-binding cassette transporters A1 and G1, HDL metabolism, cholesterol efflux, and inflammation: important targets for the treatment of atherosclerosis. Curr Drug Targets.

[39] Mathur P, Ostadal B, Romeo F, Mehta JL (2015). Gender-related differences in atherosclerosis. Cardiovasc Drugs Ther.

